# Revisiting the mechanics and energetics of walking in individuals with chronic hemiparesis following stroke: from individual limbs to lower limb joints

**DOI:** 10.1186/s12984-015-0012-x

**Published:** 2015-02-27

**Authors:** Dominic James Farris, Austin Hampton, Michael D Lewek, Gregory S Sawicki

**Affiliations:** Joint Department of Biomedical Engineering, University of North Carolina – Chapel Hill & North Carolina State University, EB 3, 911 Oval Drive, Raleigh, 27965-7115 USA; School of Human Movement & Nutrition Sciences, The University of Queensland, Human Movement Studies Bldg, Blair Drive, St Lucia, QLD 4072 USA; Division of Physical Therapy, Department of Allied Health Sciences, University of North Carolina, 3043 Bondurant Hall, CB# 7135, Chapel Hill, NC 27599-7135 USA; Human Movement Science Program, University of North Carolina Chapel Hill, Chapel Hill, USA

**Keywords:** Locomotion, Individual limbs method, Mechanical power, Metabolic power, Joint power, Inverse dynamics, Stroke

## Abstract

**Background:**

Previous reports of the mechanics and energetics of post-stroke hemiparetic walking have either not combined estimates of mechanical and metabolic energy or computed external mechanical work based on the limited combined limbs method. Here we present a comparison of the mechanics and energetics of hemiparetic and unimpaired walking at a matched speed.

**Methods:**

Mechanical work done on the body centre of mass (COM) was computed by the individual limbs method and work done at individual leg joints was computed with an inverse dynamics analysis. Both estimates were converted to average powers and related to simultaneous estimates of net metabolic power, determined via indirect calorimetry. Efficiency of positive work was calculated as the ratio of average positive mechanical power ${\overline{P}}^{+}$ to net metabolic power.

**Results:**

Total ${\overline{P}}^{+}$ was 20% greater for the hemiparetic group (H) than for the unimpaired control group (C) (0.49 vs. 0.41 W · kg^−1^). The greater ${\overline{P}}^{+}$ was partly attributed to the paretic limb of hemiparetic walkers not providing appropriately timed push-off ${\overline{P}}^{+}$ in the step-to-step transition. This led to compensatory non-paretic limb hip and knee ${\overline{P}}^{+}$ which resulted in greater total mechanical work. Efficiency of positive work was not different between H and C.

**Conclusions:**

Increased work, not decreased efficiency, explains the greater metabolic cost of hemiparetic walking post-stroke. Our results highlighted the need to target improving paretic ankle push-off via therapy or assistive technology in order to reduce the metabolic cost of hemiparetic walking.

## Background

The physics of human walking has been well described by dynamic walking models that represent the stance limb as an inverted pendulum and the swing limb as a normal pendulum during single support [1-4]. Critical in these models is the transition from one inverted pendulum to the next that requires a redirection of the centre of mass (COM) velocity from forward and downward at the end of a step to forward and upward early in the next step. In this transition, the trailing limb does positive push-off work accelerating the COM into the next step. Simultaneously, the leading limb does opposing negative work on the COM as it contacts the ground and exerts a braking force. This negative work is referred to as ‘collision’ work. Overall mechanical work done during a gait cycle is minimised when the push-off is initiated prior to collision and push-off work is approximately equal in magnitude to collision work [1,5]. This scenario minimises the work that must be done outside of the transition to maintain constant speed. Minimising the mechanical work for walking is desirable as it reduces the mechanical and metabolic energy demands of the musculoskeletal system.

Applications of these simple models to analyses of unimpaired gait have been made via the individual limbs method (ILM) [6]. This method computes the contributions of each leg to COM power individually, rather than computing the net contribution of both as is done for the combined limbs method (CLM). Thus, the ILM has facilitated a better understanding of the different roles of the leading and trailing limbs during double support. ILM analyses have revealed that unimpaired persons transition from one step to the next by applying almost equal amounts of positive push-off work with the trailing limb and negative collision work with the leading limb, simultaneously [6]. However, a common characteristic of hemiparetic post-stroke walking is a lack of ability to provide push-off work with the paretic limb (PL) [7-9]. Transition theories suggest that a lack of positive push-off work will increase negative collision work and, in turn, increase positive work done during single support to compensate [1]. This could explain the overall increase in positive mechanical work and concurrent increases in metabolic rate observed for post-stroke walking by Detrembleur and colleagues [10] when these authors compared their data to values for unimpaired individuals walking at similar speeds. Thus, transition focussed experimental analyses (i.e. the ILM) could provide an important perspective on the altered mechanics of gait after stroke and how these mechanics are linked to the elevated metabolic cost of walking post-stroke.

Whilst transition mechanics could provide useful insight into why post-stroke walking incurs an elevated mechanical and metabolic cost [10], they do not identify which joints within the limb exhibit reduced push-off work or provide the compensatory single support work. In healthy walking the ankle plantar-flexors are responsible for 40-50% of total positive work over a gait cycle, most of which is done during push-off [11]. Studies of lower limb net joint mechanics during post-stroke walking indicate that the paretic plantar-flexors of post-stroke walkers do not generate ankle push-off power comparable to those of unimpaired controls [7,8]. This observation is supported by results of dynamic computer simulations [12]. Both simulations and experiments indicated that the lack of ankle push-off power was compensated for by work done by hip musculature. Interestingly, it has been postulated that work done by hip musculature is done less efficiently than work done by ankle musculature, owing to hip muscles being less able to exploit elastic energy storage and return in series elastic structures [13]. Therefore, the observed shift in mechanical work production from the ankle to the hip could make post-stroke walking less efficient, incurring a greater metabolic cost penalty than would be observed with increased work alone.

The reduced muscular efficiency hypothesis is not supported by studies [10,14] that have determined the efficiency of post-stroke walking using mechanical work quantities calculated by the combined limbs method (CLM) [6,15]. Detrembleur et al. [10] found no difference in efficiency of walking (rate of net positive mechanical work / rate of net metabolic energy consumed) between healthy and post-stroke gait. However, by calculating total positive COM work based on the CLM, the effects of altered transition or joint mechanics on total positive work could have been masked and these studies may have underestimated muscular work for post-stroke walking [6,16]. The joint power method (JPM) of analysing lower limb mechanics computes the average power contributions made at each joint (ankle, knee and hip) and thus reveals more information about where power is being generated [11]. Experiments linking ILM and JPM-based mechanics of post-stroke walking to metabolic energy costs are required to better understand how mechanics and energetics of post-stroke gait are related.

Therefore, in this study we utilised both the JPM and ILM in conjunction with measures of metabolic energy consumption to provide a novel perspective on the relation between mechanics and energetics in post-stroke walking compared to healthy controls. We aimed to link the mechanical differences between post-stroke and speed-matched unimpaired walking to their respective metabolic costs. Matching speeds required that unimpaired controls were walking at relatively slow speeds, well below their metabolic optimum. However, this allowed the effects of altered mechanics on metabolic costs to be isolated from those of speed, which is a covariate. Walking mechanics were analysed using the ILM and JPM approaches. We hypothesised that: 1) Post-stroke walkers would exhibit reduced push-off work by the trailing paretic limb owing to lesser ankle joint work. 2) Reduced paretic trailing limb push-off work would result in increased non-paretic leading limb negative collision work for post-stroke walkers. 3) Post-stroke walkers would compensate for increased collision work by generating more work at the hip of the non-paretic limb during single support than unimpaired controls do. 4) Reliance on positive work generated at the hip would increase total positive mechanical work and reduce efficiency of work for post-stroke walkers.

## Methods

### Experimental protocol

Eight individuals with post-stroke hemiparesis (H) [six males and two females (mean ± s.d. age = 58 ± 11 years; mass = 95 ± 19 kg; height = 1.77 ± 0.06 m; time post-stroke = 9 ± 8 years)] and ten unmatched unimpaired controls (C) [six males and four females (mean ± s.d. age = 25 ± 5 years; mass 72 ± 13 kg; height = 1.69 ± 0.16 m)] gave written informed consent to participate in this study. All procedures were approved by the UNC-Chapel Hill institutional review board. Additional descriptors for the H group are provided in Table 1.

**Table 1:** **Additional participant information for the hemiparetic walkers**

**Participant**	**Age (years)**	**Mass (kg)**	**Years post-stroke**	**Preferred over-ground walking speed (m⋅s** ^**−1**^ **)**
1	54	90	28	1.02
2	45	69	5	0.52
3	49	80	4	0.74
4	56	82	4	1.15
5	67	119	10	0.78
6	80	90	9	0.62
7	56	106	1	0.88
8	55	121	7	1.10
Mean ± s.d.	58 ± 11	95 ± 19	9 ± 8	0.85 ± 0.23

Each participant walked on a split-belt instrumented treadmill (Bertec, USA) for four minutes at 0.75 m · s^−1^. Our reasons for choosing this set speed were: 1. To control for any confounding effects of speed; 2. It fell in the mid-range of preferred speeds for H; 3. All participants could maintain this speed for long enough to make steady-state metabolic measurements. To prevent falling in the event of a trip, the H group wore a harness that provided no weight support while they were walking. Participants were discouraged from using the handrails of the treadmill other than for small balance corrections if needed.

### Metabolic measurements

Rates of oxygen consumption and carbon dioxide production were recorded using a portable metabolic system (Oxycon Mobile, VIASYS Healthcare, USA). Prior to walking trials, measurements were made during five minutes of quiet standing and values from the last two minutes were averaged and used to calculate rates of metabolic energy consumption (watts) whilst standing. For the walking trials, data from the last two of the four minutes were averaged for the calculation of metabolic rate. Visual inspection of rates of oxygen consumption with time (averaged over 30 s intervals) confirmed that participants were at steady-state during this period. The respiratory exchange ratio never exceeded 1.0. Rates of oxygen consumption and carbon dioxide production were converted to metabolic powers using standard equations detailed by Brockway [17]. Net metabolic power during walking was calculated by subtracting metabolic power during standing from metabolic power during walking and these values were normalized to individual body mass (W · kg^−1^).

### Kinematics and kinetics

An eight-camera motion analysis system (Vicon, Oxford, UK) was used to capture (120 Hz) the positions of 37 reflective markers attached to the pelvis and legs (modified Cleveland Clinic marker set) of the hemiparetic participants. The marker set consisted of clusters of markers on each segment and anatomical markers placed over: right and left anterior-superior iliac spines; right and left posterior-superior iliac spines; medial and lateral femoral epicondyles; medial and lateral malleoli; calcaneus and the first and fifth metatarsal-phalangeal joints. Clusters of three or four markers on rigid plates were attached to the pelvis, thigh and shank segments to track segment motion during walking. For the feet, a cluster of three markers was attached directly to each of the participant’s shoes. Raw marker positions were filtered using a second order low-pass Butterworth filter with a cut-off frequency of 10 Hz. A static standing trial was captured and the positions of anatomically positioned markers on segments were used to calibrate a seven segment (pelvis, thighs, shanks and feet) model for each subject using established inertial parameters [18] and modelling segments as geometric cones or cylinders. The calibration process scales the segment masses, dimensions and inertial parameters to match the anthropometrics of the individual, using the anatomical reference markers listed above. Models were generated in Visual 3D software using the default segment geometries (C-motion Inc., USA) and had six degrees of freedom (three translational and three rotational) between all segments. Joint angles for the hip, knee and ankle were computed in three dimensions as the orientation of the distal segment with reference to the proximal segment and differentiated to calculate joint velocities. The same process was used to obtain kinematic data from the unimpaired controls but for the right leg and pelvis only (see [11] for details). Therefore, joint-level kinematic and inverse dynamics data for the control participants was only computed from the right leg and the left leg was assumed to behave symmetrically and out of phase by 50% of a stride. For ease of comparison in figures and tables, this paper will refer to right and left legs for the control group although the left leg data is data from the right leg shifted by 50% of stride time. However, statistical comparisons were all made between paretic limb, non-paretic limb and right control limb during comparable phases in the gait cycle.

Ground reaction force data were recorded during walking by the force sensors embedded in the treadmill (sampled at 980 Hz by the Vicon system). Participants were required to walk with each foot hitting its ipsilateral force platform, so as to separate out individual limb contributions during double support. Raw analogue force platform signals were filtered with a second order low-pass Butterworth filter with a cut-off frequency of 35 Hz. Inverse dynamic analyses were then used to compute net joint moments for all three rotational degrees of freedom at each joint, which were multiplied with their respective joint angular velocities to calculate joint powers at the hip, knee and ankle. Thus, these powers did not include the contributions of the translational degrees of freedom. This approach was adopted because the rotational contributions account for over 80% of total positive work [19] and the translational components have low signal to noise ratios (e.g. due to movement artefact). Joint kinematics and kinetics were calculated using Visual 3D software (C-motion Inc., USA).

Three-dimensional ground reaction forces (GRF) were used to compute COM velocity assuming that gait was periodic as has been described in detail previously [6,15]. Briefly, net forces acting on the COM were divided by body mass to compute COM acceleration. COM acceleration was integrated and treadmill belt speed was added to the fore-aft component to obtain COM velocity. Steady-state hemiparetic walking may not be periodic over steps but, should be over strides and thus, this assumption is still valid and the calculations of Donelan et al. [6] were adjusted to account for this. The COM velocity data were used to determine the timing of step-to-step transitions as described by Adamczyk and Kuo [20]. This method determines the start and end of transitions as the two time points surrounding the double support phase that exhibit the greatest angle between the sagittal plane COM velocity vector (i.e. when COM redirection starts and ends). The time points were constrained to be within 250 ms of heel strike and contralateral toe off, respectively. It is important to note that transitions do not have to coincide with heel strike and toe-off gait events. Instantaneous COM power generated by each leg was calculated as the dot product of that leg’s GRF vector and the COM velocity as per the ILM [6].

To quantify the mechanical contribution of each limb and each joint within the limbs, we calculated average positive $\left({\overline{P}}^{+}\right)$ and average negative $\left({\overline{P}}^{-}\right)$ mechanical power (synonymous with average rate of mechanical work) over specific phases of the gait cycle. The phases of the gait cycle were: 1) An entire stride - heel strike to ipsilateral heel strike. 2) Step-to-step transition - from the start of a transition to the end of that transition. For H in particular there is an important distinction between the transitions where the paretic limb (PL) was leading and where the non-paretic limb (NPL) was leading. 3) Non-transition - the period between one transition ending and the next one starting. The average power computation via the ILM and JPM have been described in detail elsewhere [11,21]. Briefly, periods of positive and negative instantaneous power generated by each limb or each joint were integrated separately over the relevant phases of the gait cycle for 8–10 strides of each participant’s data to get total positive and negative mechanical work done in each phase. Work values were then multiplied by stride frequency to yield average mechanical powers for each limb/joint during each phase of the gait cycle. Calculating average power this way means that the average powers of each phase sum to the total average power over a stride and can intuitively be related to metabolic power. Total $\left({\overline{P}}^{+}\right)$ and $\left({\overline{P}}^{-}\right)$ were quantified according to the ILM (sum of both limb contributions) and the JPM (sum of all joint contributions). For JPM total average power, the contribution of each joint (ankle, knee and hip) to total average power summed across all joints was expressed as a percentage of the total. Efficiency of positive work during walking was estimated as total $\left({\overline{P}}^{+}\right)$ divided by net metabolic power. This was calculated using both ILM and JPM estimations of total $\left({\overline{P}}^{+}\right)$.

### Statistical analyses

For all outcome variables the mean was calculated over 8–10 strides of each participant’s data and the mean and standard deviation of individual participant averages was computed for each group (H and C). Time series data (instantaneous powers) were interpolated to 101 linearly spaced samples over each stride before means were calculated. The main outcome variables were: individual limb and individual joint $\left({\overline{P}}^{+}\right)$ and $\left({\overline{P}}^{-}\right)$ for different phases of the gait cycle; and net metabolic power. Prior to running further statistical tests, a D’Agostino-Pearson omnibus test was used to check the normality of data. To test for statistical differences in outcome variables between limbs [PL, NPL and control limb (CL)], a one-way ANOVA was used. *F*-ratios for the main effect were considered significant for *P* < 0.05. If a significant main effect was found, t-tests were used to make pairwise comparisons between limbs. For outcome variables that were not related to a limb (i.e. total mechanical average power and net metabolic power) a t-test was used to compare between H and C.

## Results

**Figure 1:**
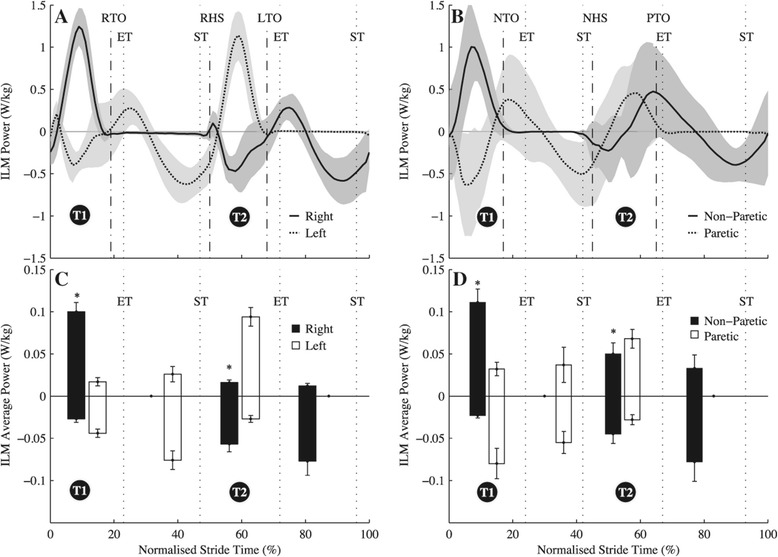
**Group mean instantaneous and average ILM powers. (A)** Group mean (± s.d.) individual limbs method (ILM) instantaneous powers for unimpaired controls, normalised to 101 points over a stride starting from left foot heel strike. **(B)** Group mean (± s.d.) ILM instantaneous powers for hemiparetic walkers normalised to 101 points over a stride starting from paretic limb heel strike. **(C)** Group mean (± s.e.m.) positive and negative average limb powers during each of the transition-based phases of the gait cycle for unimpaired controls. **(D)** Group mean (± s.e.m.) positive and negative average limb powers during each of the transition-based phases of the gait cycle for hemiparetic walkers. For **(C)** and **(D)** the bars between each of the vertical dotted lines (indicating step-to-step transition events) represent average positive or negative power generated by each limb over the period between those transition events. ST - start of step-to-step transition; ET - end of step-to-step-transition; RTO - Right toe-off; RHS - right heel-strike; LTO - Left toe-off; PTO - paretic toe-off; NTO - non-paretic toe-off; NHS - non-paretic heel-strike. T1 - first transition phase, T2 - second transition phase. *Indicates significant difference between that average power value and the corresponding average power in the opposite panel, also marked with a *(i.e. a difference between unimpaired controls and hemiparetic groups).

**Figure 2:**
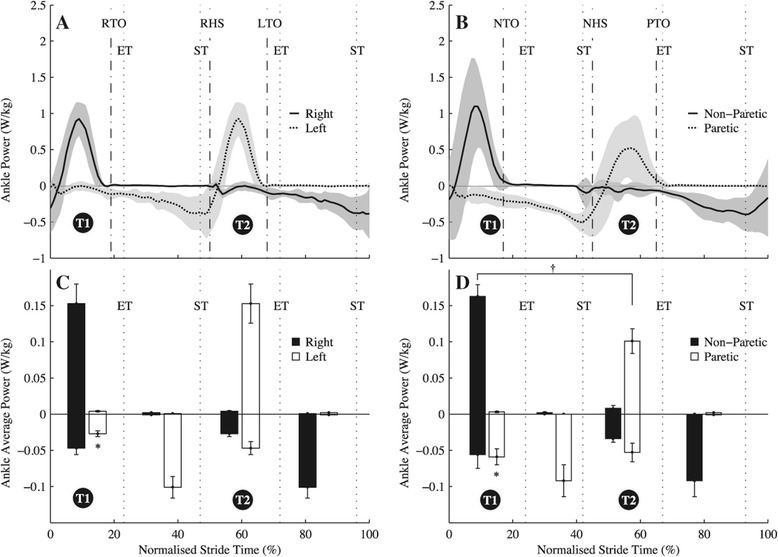
**Group mean instantaneous and average ankle joint powers. (A)** Group mean (± s.d.) Ankle instantaneous powers for unimpaired controls, normalised to 101 points over a stride starting from left foot heel strike. **(B)** Group mean (± s.d.) Ankle instantaneous powers for hemiparetic walkers normalised to 101 points over a stride starting from paretic limb heel strike. **(C)** Group mean (± s.e.m.) positive and negative average ankle powers during each of the transition-based phases of the gait cycle for unimpaired controls. **(D)** Group mean (± s.e.m.) positive and negative average ankle powers during each of the transition-based phases of the gait cycle for hemiparetic walkers. For **(C)** and **(D)** the bars between each of the vertical dotted lines (indicating step-to-step transition events) represent average positive or negative power generated by each limb over the period between those transition events. ST - start of step-to-step transition; ET - end of step-to-step-transition; RTO - Right toe-off; RHS - right heel-strike; LTO - Left toe-off; PTO - paretic toe-off; NTO - non-paretic toe-off; NHS - non-paretic heel-strike. *Indicates significant difference between that average power value and the corresponding average power in the opposite panel, also marked with a *(i.e. a difference between unimpaired controls and hemiparetic groups); + indicates significant difference between that average power value and the equivalent average power in the same panel (i.e. a difference between paretic and non-paretic ankles).

**Figure 3:**
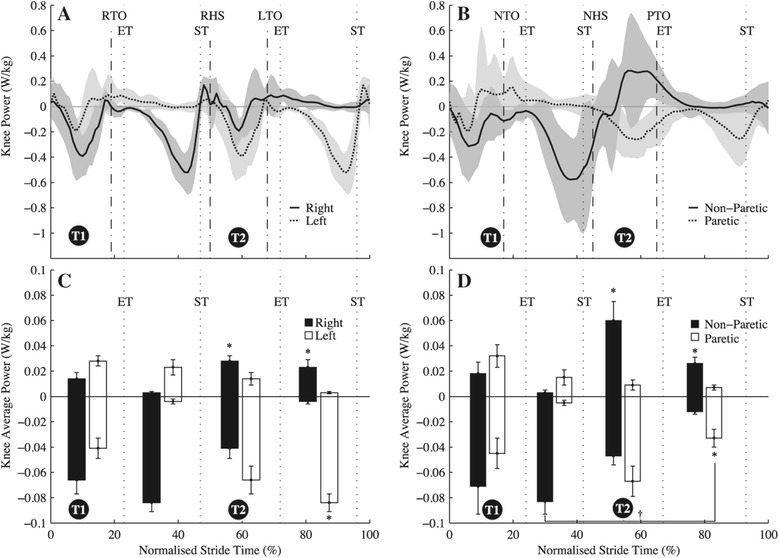
**Group mean instantaneous and average knee joint powers. (A)** Group mean (± s.d.) Knee instantaneous powers for unimpaired controls, normalised to 101 points over a stride starting from left foot heel strike. **(B)** Group mean (± s.d.) knee instantaneous powers for hemiparetic walkers normalised to 101 points over a stride starting from paretic limb heel strike. **(C)** Group mean (± s.e.m.) positive and negative average knee powers during each of the transition-based phases of the gait cycle for unimpaired controls. **(D)** Group mean (± s.e.m.) positive and negative average knee powers during each of the transition-based phases of the gait cycle for hemiparetic walkers. For **(C)** and **(D)** the bars between each of the vertical dotted lines (indicating step-to-step transition events) represent average positive or negative power generated by each limb over the period between those transition events. ST - start of step-to-step transition; ET - end of step-to-step-transition; RTO - Right toe-off; RHS - right heel-strike; LTO - Left toe-off; PTO - paretic toe-off; NTO - non-paretic toe-off; NHS - non-paretic heel-strike. *Indicates significant difference between that average power value and the corresponding average power in the opposite panel, also marked with a *(i.e. a difference between unimpaired controls and hemiparetic groups); + indicates significant difference between that average power value and the equivalent average power in the same panel (i.e. a difference between paretic and non-paretic knees).

**Figure 4:**
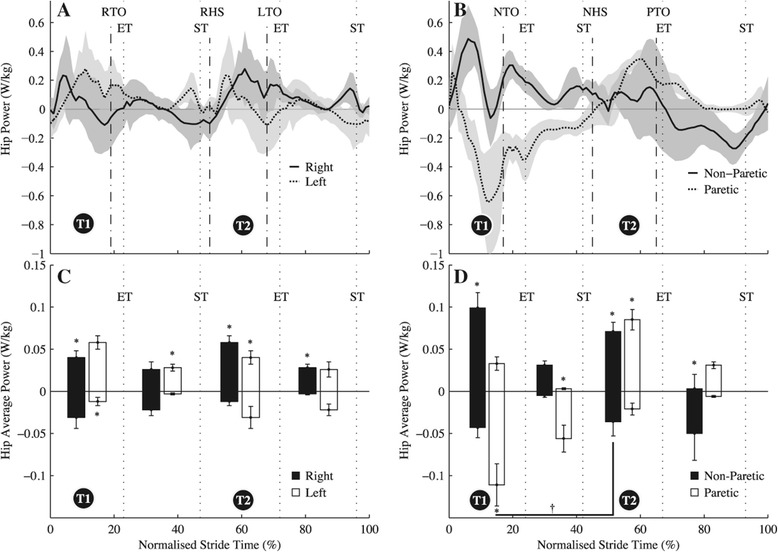
**Group mean instantaneous and average hip joint powers. (A)** Group mean (± s.d.) Hip instantaneous powers for unimpaired controls, normalised to 101 points over a stride starting from left foot heel strike. **(B)** Group mean (± s.d.) hip instantaneous powers for hemiparetic walkers normalised to 101 points over a stride starting from paretic limb heel strike. **(C)** Group mean (± s.e.m.) positive and negative average hip powers during each of the transition-based phases of the gait cycle for unimpaired controls. **(D)** Group mean (± s.e.m.) positive and negative average hip powers during each of the transition-based phases of the gait cycle for hemiparetic walkers. For **(C)** and **(D)** the bars between each of the vertical dotted lines (indicating step-to-step transition events) represent average positive or negative power generated by each limb over the period between those transition events. ST - start of step-to-step transition; ET - end of step-to-step-transition; RTO - Right toe-off; RHS - right heel-strike; LTO - Left toe-off; PTO - paretic toe-off; NTO - non-paretic toe-off; NHS - non-paretic heel-strike. *Indicates significant difference between that average power value and the corresponding average power in the opposite panel, also marked with a *(i.e. a difference between unimpaired controls and hemiparetic groups); + indicates significant difference between that average power value and the equivalent average power in the same panel (i.e. a difference between paretic and non-paretic hips).

**Figure 5:**
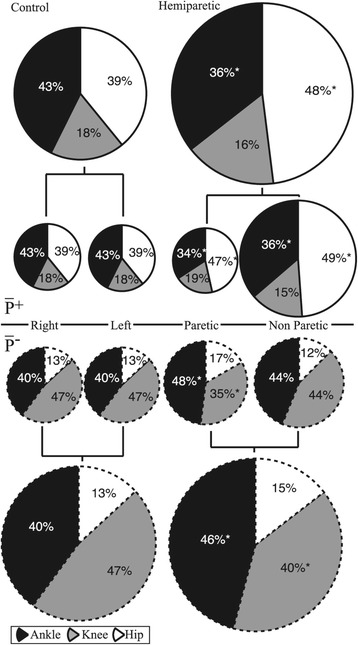
**Group mean distributions of** $\left({\overline{\boldsymbol{P}}}^{+}\right)$ **(solid outline) and** $\left({\overline{\boldsymbol{P}}}^{-}\right)$ **(dashed outline) as determined by the JPM.** Top and bottom pies for each represent the distribution when both limbs are summed. The distributions of $\left({\overline{P}}^{+}\right)$ and $\left({\overline{P}}^{-}\right)$ within each limb are then represented in the two smaller pies linked to each larger pie. The total area of each pie represents total work of that pie relative to all other pies. *denotes a statistically significant difference from unimpaired controls (*P* < 0.05).

Group mean time histories of instantaneous ILM powers over an average stride (±s.d.) are shown in Figure 1 and instantaneous joint flexion-extension powers for the ankle, knee and hip are in Figures 2, 3 and 4, respectively. Total ${\overline{P}}^{+}$ determined by the ILM was 0.27 ± 0.06 W · kg^−1^ for C and 0.33 ± 0.09 W · kg^−1^ for H (t(16) = 2.34, *p* = 0.02). By the JPM, total ${\overline{P}}^{+}$ was 0.41 ± 0.05 and 0.49 ± 0.03 W · kg^−1^ for C and H, respectively (t(16) = 2.60, *p* = 0.02). As can be seen from the t-statistic and *P* values, for both methods total ${\overline{P}}^{+}$ was significantly greater for H. Total NPL ${\overline{P}}^{+}$ was significantly greater than total PL and C limb ${\overline{P}}^{+}$ (Table 2, *p* < 0.001). Figure 1 breaks up limb ${\overline{P}}^{+}$ and ${\overline{P}}^{-}$ into transition and non-transition values. Also, Figures 2, 3 and 4 show ${\overline{P}}^{+}$ and ${\overline{P}}^{-}$ for the ankle, knee and hip in and out of transition. As can be seen in Figure 5, there was a shift in distribution of ${\overline{P}}^{+}$ for H compared to C with a significantly greater proportion of ${\overline{P}}^{+}$ being generated at the hips (48% vs. 39%, t(16) = 4.21, *p* < 0.001). Metabolic power was significantly (t(16) = 3.69, *p* = 0.003) greater for H (3.02 ± 0.27 W · kg^−1^) than for C (1.99 ± 0.06 W · kg^−1^). Efficiency of positive work was not different between H and C when estimated from the ILM (H = 0.11 ± 0.02 vs. C = 0.15 ± 0.06, t(16) = 1.42 *p* = 0.18) or the JPM (H = 0.16 ± 0.01 vs. C = 0.24 ± 0.13, t(16) = 1.37, *p* = 0.23) values for total ${\overline{P}}^{+}$.

**Table 2:** **Group mean (± s.e.m.) average positive power generated over a stride by the control limb (CL), paretic limb (PL), Non-Paretic limb (NPL) and their individual joints**

	**CL**	**PL**	**NPL**
ILM	0.14 ± 0.01	0.14 ± 0.01	0.19 ± 0.01^+^
JPM	0.38 ± 0.10	0.32 ± 0.10	0.49 ± 0.01^+^
Ankle	0.16 ± 0.05	0.11 ± 0.04	0.17 ± 0.06*
Knee	0.068 ± 0.02	0.06 ± 0.02	0.11 ± 0.04^+^
Hip	0.15 ± 0.03	0.15 ± 0.01	0.21 ± 0.07^+^

*denotes significantly different from the paretic limb (*P* <0.05).

^+^denotes significantly different from paretic and control limb (*P* <0.05).

## Discussion

### Step-to-step transitions

First we will consider the transition where the NPL was leading which is the second transition (T2) in Figures 1, 2, 3 and 4(B, D) and can be compared to the second transition (T2) in Figures 1, 2, 3 and 4(A, C). Our first hypothesis was that the PL would produce less push-off ${\overline{P}}^{+}$ than an unimpaired control limb in these transitions. This was assessed by comparing the ${\overline{P}}^{+}$ provided by each limb during transitions when it was the trailing limb (e.g. Figure 1C - T2, positive white bar for the PL). Our results did not support the hypothesis as the PL did not provide significantly less ${\overline{P}}^{+}$ than control limbs when trailing in a transition (Figure 1 - T2). This was despite peak instantaneous power during those transitions being less for the PL (PL = 0.46 W⋅kg^−1^, NPL = 1.01 W⋅kg^−1^, C = 1.19 W⋅kg^−1^, *F(2,23)* = 4.5, *p* <0.001) and indicated a more prolonged, lower magnitude period of push-off by the PL as can be observed in Figure 1. Given that our first hypothesis was not supported, it is unsurprising that our second hypothesis was also not supported by our data. We predicted that the anticipated reduction in PL push-off ${\overline{P}}^{+}$ would lead to increased negative collision ${\overline{P}}^{-}$ by the NPL in the same transition. The ${\overline{P}}^{-}$ of the NPL in transitions when it was leading was not significantly different from ${\overline{P}}^{-}$ done by control limbs when leading (Figure 1C and D - T2, negative dark bars).

Despite the magnitudes of push-off and collision average power not being significantly different between H and C during T2, the ILM power curves were markedly different from those of the unimpaired controls (Figure 1A-B). For C, the trailing left limb did positive push-off work at the same time as the leading right limb did negative collision work and this occurred over the majority of the transition (Figure 1A -T2). This serves to redirect the centre of mass so it can begin the next inverted pendulum phase, as has been described for healthy gait previously [6]. For H transitions when the NPL was leading, there was only a brief period (≈5% of a stride) when the two limbs were producing opposing powers (Figure 1B - T2). At the beginning of this transition, both limbs were generating negative COM power before the brief period when the PL provides positive power and the NPL provides negative power. The latter half of this transition involved both PL and NPL generating positive COM power (Figure 1B -T2). It was during this latter part of the transition where significant differences from controls were observed in terms of average powers. The leading NPL for H provided significantly (*F(2,23)* = 4.7, *p* = 0.009) more ${\overline{P}}^{+}$ during these transitions than the leading limb for C, that was predominantly providing negative collision work (Figure 1). Interestingly, this corresponded with greater ${\overline{P}}^{+}$ at the leading non-paretic hip (*F(2,23)* = 3.51, *p* = 0.003) and knee (*F(2,23)* = 3.11, *p* = 0.05) for H than at the leading hip and knee for C (Figures 3 and 4). Therefore it seems that H used a different temporal sequencing of limb and joint power production than C.

To interpret the effects of the altered temporal sequencing, we may gain some insight from a simple model of walking. Kuo [22] presented a passive dynamic walking model [3] with the ability to apply a trailing limb toe-off impulse just prior to heel strike or a leading limb hip torque after collision. Either could be used to redirect the centre of mass velocity in the transition between steps. Kuo [22] observed that the overall mechanical work required per step was four times greater when the pre-emptive push-off impulse was not used and hip torque following collision was relied upon to redirect the COM. In this simple model the collision occurs instantaneously and the only source of work after the collision was a hip torque. Neither of these assumptions has to be true for human gait but the model does illustrate that if push-off work is not initiated prior to or at heel strike, the positive work required to maintain walking speed must be done later in the step and is larger in magnitude. Similarly, Soo and Donelan [5] showed experimentally that deviating from preferred coordination in transitions can increase the mechanical work requirements of movement. This is relevant to the transition described above for H (where NPL is leading -T2) and might explain the need for additional ${\overline{P}}^{+}$ at the non-paretic hip and knee in these transitions. Figure 1B shows that, for a transition with the NPL leading, the push-off work from the PL was not initiated until 7 ± 2.0% of a stride after the NPL heel strike. This was significantly (*F(2,23)* = 3.4, *p* = 0.02) later after heel strike than for the control limb that initiated push-off almost at heel strike (1 ± 2.7%, Figure 1A). Also, the H group incurred a greater overall average positive power demand in the step starting with this transition than the C group did during a step (H = 0.17 W⋅kg^−1^, C = 0.135 W⋅kg^−1^, *F(2,23)* = 3.2, *P* = 0.04). This additional work came from significantly greater (compared to control) average powers generated at the knee and hip in the NPL and the hip in the PL in the transition (Figures 3 and 4 -T2). Additional ${\overline{P}}^{+}$ was also generated at the non-paretic knee after the transition was completed (Figure 3B,D). Our third hypothesis was that the non-paretic hip would provide additional ${\overline{P}}^{+}$ to meet the added work demands of this step (beginning with T2). This was supported but the non-paretic knee and the paretic hip also contributed to the additional work requirement in this step for H (Figures 3 and 4).

No hypotheses were made regarding the transition in which the PL was leading (the first transition, T1, in Figures 1, 2, 3 and 4) because it was expected that the NPL would be capable of providing push-off power comparable to healthy limbs. Indeed, the non-paretic trailing limb was capable and actually provided significantly greater ${\overline{P}}^{+}$ than control trailing limbs during the transition (Figure 1 - T1). This ${\overline{P}}^{+}$ increase was mostly owing to increases in hip average positive power in the NPL at this time (Figure 4). The reason for this additional positive work seems to have been to counteract a larger amount of collision ${\overline{P}}^{-}$ that was simultaneously being provided by the hip and ankle joints of the paretic leading limb, compared to control leading limbs (Figures 2 and 4). Based on the current data we were unable to provide an explanation for increased simultaneous positive and negative average power during this transition compared to C. Step length was not different and the timing of push-off was near optimal for the NPL (2.0 ± 4.9% after heel-strike). Stroke survivors commonly display impaired motor control [23] in addition to muscle weakness [8] and so perhaps the explanation is related to poor control of the movement. Thus the greater collision work might represent a limited ability to stabilize the leading PL against gravity during weight acceptance and the additional NPL positive work was a pre-emptive compensation, but this is speculation. Regardless of the reasoning, this large collision contributes to the overall increase in positive mechanical work required by H.

### Distribution of positive work

As was expected from previous reports [10,14] of the external work requirements of hemiparetic gait, total ${\overline{P}}^{+}$ was greater for H than C. This increased demand was met by greater ${\overline{P}}^{+}$ from the NPL compared to the CL (Table 2). The PL provided similar ${\overline{P}}^{+}$ to the CL (Table 2). These findings were independent of what method (ILM or JPM) was used to quantify total ${\overline{P}}^{+}$. Summing joint average powers will show large discrepancies in absolute total ${\overline{P}}^{+}$ values compared to the ILM. This is to be expected as cancellations of work occur internally within the limb when two joints do simultaneous opposing work, leading to the ILM underestimating total ${\overline{P}}^{+}$ [16]. This section will focus on the values determined via the JPM.

Our third and fourth hypotheses both predicted that the increased total positive work demand for H would be met by an increase in non-paretic hip work compared to C. As one might anticipate from the prior description of transition work, hip ${\overline{P}}^{+}$ was greater for the NPL than both the PL and CL (Table 2). This led to the non-paretic hip being responsible for 49% of the ${\overline{P}}^{+}$ provided by the NPL compared to 39% for C hips (Figure 5). The PL also relied on the hip joint ${\overline{P}}^{+}$ generation more than CL (47% vs. 39%, Figure 5). Therefore, in addition to H having to generate greater overall ${\overline{P}}^{+}$, they also redistributed ${\overline{P}}^{+}$ among joints to rely more on the hip than C. This agrees well with previous inverse dynamics-based studies of imposed ankle immobility during walking in healthy controls [5,24] and hemiparetic post-stroke gait [8]. This supported the rationale for our final hypothesis regarding efficiency of positive mechanical work.

### Efficiency of positive work

Metabolic power was 52% greater for hemiparetic individuals than it was for the unimpaired controls. This was to be expected given that total $\left({\overline{P}}^{+}\right)$ was significantly greater (Table 1). In our fourth hypothesis we proposed that the metabolic power increase for H would be greater than that expected from the increased mechanical work alone. This was rationalised by the theory that the shift to greater reliance on the hip for mechanical power would make locomotion less efficient [13]. This prediction was not supported by the efficiency data that showed no significant difference in efficiency of positive work between H and C. On a cautionary note, the efficiency data had low statistical power and therefore we cannot with complete certainty reject the hypothesis. If the present result does hold true for a larger population, one plausible explanation for this is that slow walking is not very efficient for the C group. Walking at 0.75 ms^−1^ is less efficient than walking at faster, more optimal speeds for unimpaired humans (0.26 vs. 0.34 [11]). In this study the hypothesised decrease in efficiency was proposed to be due to reliance on less efficient hip musculature more than on efficient ankle plantar-flexors. However, since the efficiency of control walking at 0.75 ms^−1^ seems to be similar to what one would expect from hip muscle anyway [13], the rationale based on distribution of work no longer holds for this speed. The matched-speed study design employed allowed this finding to be highlighted and showed that mechanics associated with post-stroke gait can increase the metabolic cost of locomotion without necessarily making individuals less efficient than unimpaired controls walking at the same speed.

### Limitations

There were some limitations to our study design. First, the controls were not matched for age with the stroke survivors. Therefore we cannot conclusively reject the possibility that some differences between the two groups were related to effects of ageing. As has been observed by Franz and Kram [25], older individuals exhibit reduced trailing limb push off work during level walking compared to younger controls and this is compensated for in single support later in the step. However, these authors also showed that total work over a gait cycle was not significantly different during level walking between old and young individuals and the older individuals utilised similar timing and trajectories for COM mechanics even though some magnitudes were different. In contrast, our key findings for the hemiparetic group were that they exhibited altered timing of push off and collision work; asymmetrical mechanics and a resulting increased overall rate of mechanical work in comparison to younger healthy controls. Furthermore, the older adults in Franz and Kram [25] were notably older than the majority of our hemiparetic individuals (72 ± 5 vs. 58 ± 11 years) although there was one notable exception at 80 years of age (participant 6, Table 1) whose data may have been more affected by age than others. Therefore, although there is some potentially confounding effect of age, we maintain that our comparison highlights altered walking mechanics that result from hemiparesis that have not been observed as a result of aging. Furthermore, our findings related to efficiency and mechanical work done on the COM were consistent with previous comparisons of matched unimpaired post-stroke cycling and walking [9,26].

Another limitation was that we did not control the level of impairment of the stroke survivors included in the study beyond them needing to be able to walk unassisted on the treadmill at 0.75 m⋅s^−1^. This may explain some of the large standard deviations observed for H. The study employed matched walking speed for the control group with the aim of examining the effects of altered mechanics, independent of speed. However, as noted previously, this forces the control group away from their most efficient and preferred walking speeds. Therefore, care should be taken not to extrapolate the findings to comparisons of walking mechanics and energetics for self-selected speeds between post-stroke and unimpaired walking. A final limitation was that sample sizes were small, especially for H. Results of a post-hoc statistical power analysis performed with G*Power software v3.1 [27] are shown in Table 3. Overall, statistical power values were greater than 0.97 for most mechanical variables and metabolic power. However, for efficiency data power was low (0.54). Thus we cannot have complete confidence in rejecting the possibility that the hemiparetic group walked less efficiently than the controls in this study.

**Table 3:** **Results of statistical power analysis from exemplar statistical tests**

**Variable**	**Test**	Effect Size*	**Power**
Metabolic Power	t-test	5.26	1.00
Total $\left({\overline{P}}^{+}\right)$ from JPM	t-test	1.94	0.98
Efficiency from JPM	t-test	0.87	0.54
Total $\left({\overline{P}}^{+}\right)$ from ILM	t-test	0.78	0.48
Efficiency from ILM	t-test	0.89	0.56
Limb $\left({\overline{P}}^{+}\right)$ from ILM	ANOVA	0.87	0.97
Limb $\left({\overline{P}}^{+}\right)$ from JPM	ANOVA	1.1	0.99
Ankle $\left({\overline{P}}^{+}\right)$	ANOVA	2.02	1.00

*Effect sizes are Cohen’s d for t-tests and f for ANOVAs.

### Applications

The inability of H to produce appropriately timed push-off power with their PL and the subsequent necessary compensations highlighted the importance of targeting this phase of the gait cycle with interventions to rehabilitate or facilitate locomotion. It was not clear whether the deficit rests more with impaired control or weakened plantar-flexor muscles but interventions to restore function in this muscle group have the potential to reduce overall muscle work and metabolic cost in post-stroke walking. Furthermore, in the case that assistance is required, devices that can provide appropriately timed ankle plantar-flexion power may also reduce mechanical work and metabolic cost. Particularly portable devices utilizing optimally sized springs in parallel with the limb joints to help control the paretic limb collision by capturing excess negative work early in stance and then returning it to supply a more impulsive paretic push-off may be appropriate (e.g. [28-30]).

## Conclusions

In this study we compared the mechanics and energetics of post-stroke hemiparetic walking and speed-matched unimpaired control walking. We concluded that suboptimal timing of paretic limb push-off resulted in an increased work requirement for hemiparetic individuals. This increased demand was met by generating more positive work at the non-paretic hip and knee, and the paretic hip. This incurred a significantly greater metabolic cost without affecting the efficiency of positive mechanical work. We propose that restoring appropriate ankle push-off timing for the paretic limb has potential to reduce mechanical and metabolic demands in post-stroke walking. This may be achieved through therapy or with assistive devices [28,29,31].
